# Reduced variability of bursting activity during working memory

**DOI:** 10.1038/s41598-022-18577-y

**Published:** 2022-09-05

**Authors:** Mikael Lundqvist, Jonas Rose, Scott L. Brincat, Melissa R. Warden, Timothy J. Buschman, Pawel Herman, Earl K. Miller

**Affiliations:** 1 Department of Psychology, Department of Clinical Neuroscience, Karolinska Institute, Solna, Sweden; 2 The Picower Institute for Learning and Memory and Department of Brain and Cognitive Sciences, Massachusetts Institute of Technology, Cambridge, MA 02139 USA; 3 Faculty of Psychology, Neural Basis of Learning, Ruhr University Bochum, 44801 Bochum, Germany; 4 Department of Neurobiology and Behavior, Cornell University, Ithaca, NY 14853 USA; 5 Princeton Neuroscience Institute, Princeton University, Washington Rd., Princeton, NJ 08540 USA; 6 Department of Computational Science and Technology, School of Electrical Engineering and Computer Science and Digital Futures, KTH Royal Institute of Technology, 100 44 Stockholm, Sweden

**Keywords:** Neuroscience, Cognitive neuroscience, Computational neuroscience, Learning and memory, Neuronal physiology

## Abstract

Working memories have long been thought to be maintained by persistent spiking. However, mounting evidence from multiple-electrode recording (and single-trial analyses) shows that the underlying spiking is better characterized by intermittent bursts of activity. A counterargument suggested this intermittent activity is at odds with observations that spike-time variability reduces during task performance. However, this counterargument rests on assumptions, such as randomness in the timing of the bursts, which may not be correct. Thus, we analyzed spiking and LFPs from monkeys’ prefrontal cortex (PFC) to determine if task-related reductions in variability can co-exist with intermittent spiking. We found that it does because both spiking and associated gamma bursts were task-modulated, not random. In fact, the task-related reduction in spike variability could largely be explained by a related reduction in gamma burst variability. Our results provide further support for the intermittent activity models of working memory as well as novel mechanistic insights into how spike variability is reduced during cognitive tasks.

## Introduction

Working memory (WM), the holding of information “online” in a form available for processing, is central in human cognition. It has long been thought that WMs are carried by persistent spiking of neurons that maintain “attractor states”^1–4^. But recent evidence suggests that WM-related activity is more dynamic^5–11^. Advances in multiple-electrode technology have allowed sampling of larger populations of neurons as well as a closer examination of their activity in real time. This revealed that the spiking activity of many neurons does not seem persistent. Instead, it is organized in intermittent bursts, especially when activity is examined on individual trials^5,6,12,13^. Because burst times vary from trial to trial, averaging across trials can create the illusion of persistent spiking when the underlying activity is intermittent^5^. Further, the intermittent spiking is not just a property of individual neurons. The bursts of spiking are associated with bursts of gamma-band power^5,6^. This suggests that the intermittent bursting is coordinated in local networks. Thus, persistence cannot be obtained by pooling local networks of neurons. This provides support to a class of synaptic attractor models in which short-term plasticity helps maintain attractor states in between bouts of intermittent spiking^14–17^. This is consistent with observations from EEG and FMRI studies that, for extended periods of time, information held in working memory cannot be decoded from global activity. However, when the cortex is “pinged” by a task-irrelevant stimulus or by transcranial magnetic stimulation, the network “rings” back with the information held in working memory^7–9^.

On the other hand﻿, observations that cortical spiking becomes less variable during performance of various tasks^18–23^ have led to a counterargument to intermittent spiking. For example, a recent modelling study argued against the synaptic attractor type models (and in favor of persistent spiking alone) by suggesting that intermittent bursting should cause an increase, not a decrease, in across-trial variability of spiking (measured by Fano Factor, FF)^24^. These arguments rest on several assumptions that may not be consistent with experimental observations. Most importantly, the model assumed that intermittent bursts of spiking occurred at random times during a WM task. However, experimental observations show that WM-related bursts of activity are not random, they wax and wane with different trial events^5,6^. This raises the question of whether this task-related modulation of bursts is enough to explain the task-related reduction in spike variability that is often observed in cortex.

To address this, we analyzed actual spiking and LFPs from the prefrontal cortex of non-human primates (NHPs) performing three working memory tasks. We previously reported intermittent bursts of spiking and associated bursts of gamma power during performance of the tasks^5,6^. Here, we report that variability of spiking (FF) decreased, not increased, during task performance. In fact, the largest task-related reduction in variability was found at recording sites where the spike rates and burst rates increased the most. We also gained insight into why variability was reduced. We synthesized spikes based on the gamma bursts found in the LFPs. This revealed that the reduction in spiking variability was directly related to reductions in gamma burst variability. These results provide further support for synaptic attractor type models of working memory with intermittent activity^15–17,25–27^.

## Results

### Increased bursting co-exists with reduced spike variability

We analyzed LFP power and spiking from multiple single neurons recorded from the lateral prefrontal cortex of monkeys performing three different WM tasks. From these data, we previously reported task-related intermittent spiking and associated bursts of gamma power^5,6^. We further demonstrated that spikes associated with the gamma bursts carried more WM information than spiking without gamma bursts. Here, we examine whether the variability of this activity increases or decreases during task performance.

**Figure 1:**
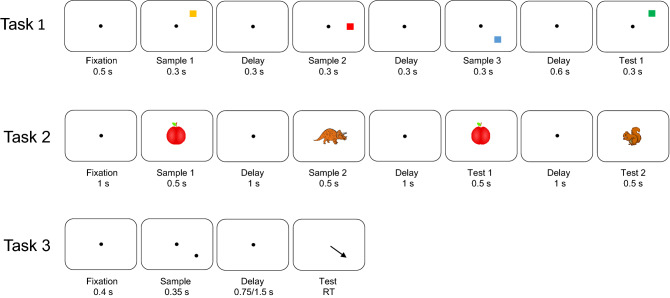
Task structure of the 3 working memory tasks. *Top*: Structure of Task 1. The monkeys were presented with a sequence of 3 colored squares in 3 distinct positions. After a brief delay, the monkeys were exposed to a new sequence of up to 3 squares. They reported with a saccade to the first square that had changed color between sample and test sequences (one of the squares always changed, in the above case the first). *Middle:* Structure of Task 2. The monkeys were presented with a sequence of 2 objects foveally. After a brief delay, the monkeys were exposed to a new sequence of 2 objects. They reported by lifting a bar if the test sequence did not match the sample sequence. *Bottom:* Structure of Task 3. Delayed saccade task with 2 possible delay lengths. On 6/7 trials, there was a 0.75 s delay, on the rest there was a 1.5 s delay.

In the tasks, monkeys had to hold 1–3 stimuli in WM. In Task 1, the animals had to remember the color and location of 3 squares presented in their visual periphery (Ref.^5^; Fig. 1). In Task 2, the animals remembered the identity and order of two foveally presented pictures (Refs.^28,29^; Fig. 1).

**Figure 2:**
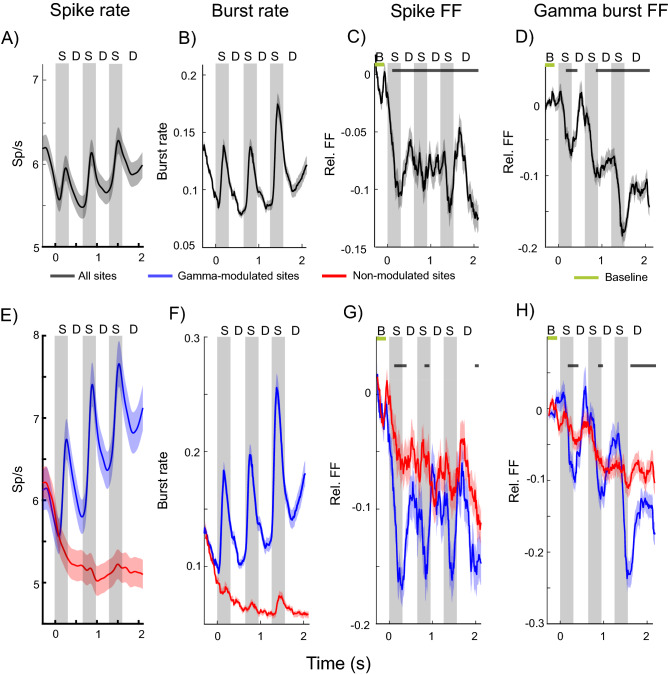
Spike and burst variability in Task 1. (**A**) Spike rates averaged across trials and all neurons (n = 495). Gray shaded regions indicate sample presentations (S; the first sample is presented at time = 0). The three delays are marked by “D”. Error bars indicate standard error of the mean in all panels. (**B**) Gamma burst rates averaged across trials for all recording sites (n = 319). (**C**) Relative FF in spike times, i.e. normalized by the pre-stimulus baseline (marked by “B”; from 300 to 100 ms prior to first sample onset) average. (**D**) Relative FF in burst times (normalization as in (**C**). Black bars indicate time periods when post stimulus FF deviated significantly from the pre-stimulus baseline (marked by the green bar, p < 0.0001, permutation-based cluster statistics^49^). (**E–H**) same as (**A–D**) but separately for sites that increased (blue; n = 145 sites and n = 214 neurons) gamma bursting at sample onset, and sites that did not (red; n = 174 sites and n = 281 neurons). Black bars indicate time periods when blue and red curves are significantly different (p < 0.03 permutation-based cluster statistics).

**Figure 3:**
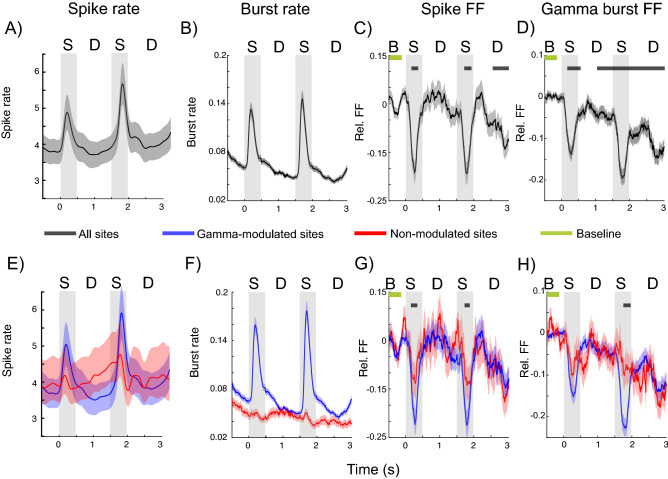
Spike and burst variability in Task 2. (**A**) Spike rates averaged across trials and all neurons (n = 283). Gray shaded regions indicate sample presentations (S; the first sample is presented at time = 0). The two delays are marked by (**D**). Error bars indicate standard error of the mean in all panels. (**B**) Gamma burst rates averaged across trials for all recording sites. (**C**) FF in spike times normalized by the pre-stimulus baseline (marked by “B”; from 500 to 100 ms prior to first sample onset) average. (**D**) FF in burst times normalized by the pre-stimulus (from 500 to 100 ms prior to first sample onset) average. (**E–H**) Same as (**A–D**) but separately for sites that increased (blue) gamma bursting at sample onset, and sites that did not (red).

Spiking and gamma burst rates were modulated by the tasks. Figure 2A shows the average spike rate as a function of time during the trial of Task 1. The gray shaded areas show the time of presentation of three to-be-remembered stimuli. As previously demonstrated, spike rate was high during the pre-stimulus baseline and dropped before presentation of the first stimulus. Presentation of each stimulus caused a transient increase in spiking. There was a ramp-up of spiking over the final memory delay just prior to test stimulus onset. Figure 2B shows that gamma burst rate follows a similar profile. Figure 3A,B shows similar results for Task 2.

**Figure 4:**
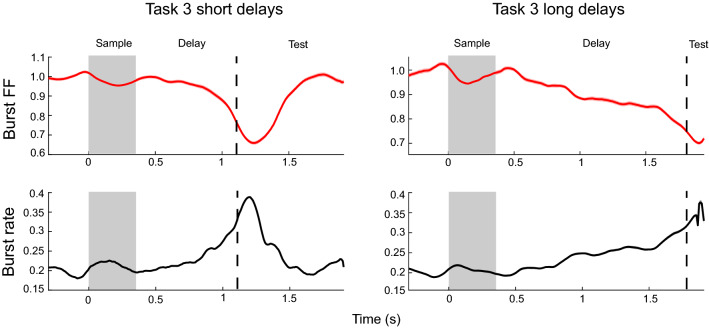
Burst variability in Task 3. Top row shows the gamma burst FF for short (left) and long (right) trials. Bottom row plots the corresponding gamma burst rates. Shaded areas indicate standard error of mean.

This modulation of spiking and gamma bursting by the task resulted in a decrease in across-trial variability. This can be seen in Fig. 2C,D (and Fig. 3C,D for Task 2), respectively, which plot the change in Fano Factor (FF) relative to the pre-stimulus baseline over the trial duration. The FF was high during the baseline and decreased during task performance. Importantly, both FFs show opposite trends to spike and gamma burst rates (c.f. Fig. 2A,B). The FFs decrease during stimulus presentations, increase between stimulus presentations, and decrease towards the end of the delay. Notably, both the spike and gamma burst FFs were lower during the memory delay than during the pre-stimulus baseline. We found similar results in spatial delayed saccade task (Task 3, Fig. 4).

Our prior work showed a relationship between gamma bursts and WM information in spiking. We found that stimulus-driven gamma bursting was limited to a subset of recording sites (145/319 = 45% in Task 1, 151/199 = 73% in Task 2; sites with significant increase in bursts during any of the stimulus presentations relative to the corresponding pre-stimulus period, using paired t-test for increase in number of bursts in the pre-stimulus to stimulus presentation time windows at threshold p = 0.05, see also “Materials and methods” section and Refs.^5,6^). We referred to these sites as “gamma-modulated sites” while the sites without stimulus-induced gamma bursting were referred to as “non-modulated sites”^5,6^. We then demonstrated that spiking at gamma-modulated sites exclusively carried information about the stimuli held in WM.

Both gamma-modulated and non-modulated sites showed a reduction in gamma-burst variability during the task. Figure 2 shows the difference in spike rates (Fig. 2E) and gamma burst rates (Fig. 2F) between gamma-modulated and non-modulated sites. Much like the average across all sites (Fig. 2B), gamma burst rate at gamma-modulated sites increased during stimulus presentation and ramped up over the memory delay (Fig. 2F, blue lines). As expected, there was less gamma bursting and no stimulus dependent modulation at non-modulated sites (Fig. 2F, red lines), and the spike rates were lower at non-modulated sites (Fig. 2E).

**Figure 5:**
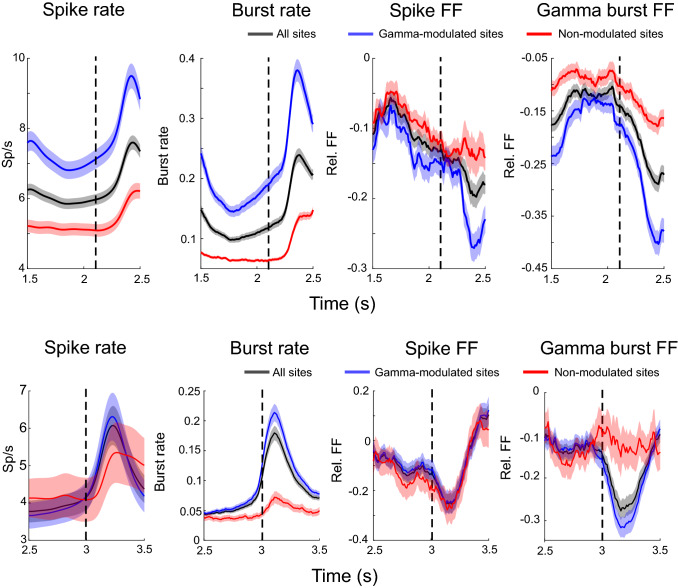
Burst and spike variability during test stimulus. Same as in Figs. 2 and 3, but focusing on the test periods for Task 1 (top) and Task 2 (bottom). Test stimulus onset is marked by dotted vertical lines in all panels.

Importantly, we found that the spiking FF showed a greater reduction at the gamma-modulated sites (Figs. 2G, 3G, blue lines) than non-modulated sites (Figs. 2G, 3G, red lines). Gamma bursting at both types of sites showed a reduction in FF as the trial progressed relative to the baseline but this effect was more pronounced at the gamma-modulated sites (Figs. 2H, 3H). During sample stimulus presentations, when both gamma burst and spike rates increased at the gamma-modulated sites, there were sharp reductions in the FFs at these sites (Figs. 2E,F, 3E,F). At test stimulus presentations, when the burst rates were at their peak, the reductions in spike and gamma burst FFs were most pronounced (Figs. 4, 5).

These analyses indicate that task modulation of spike activity and gamma bursts does result in task-related reduction in neural variability. The examination of gamma-modulated vs non-modulated sites and different task epochs further indicates that the higher the gamma bursting, the greater the reduction in both spike and gamma burst variability. The gamma bursts are thought to reflect coordinated spiking in local networks. This raises the question of whether the task-modulation of the gamma bursts can, in turn, explain the task-related reduction in spike variability. We address this below focusing on Task 1.

### Reductions of gamma burst variability help explain the reduction in spike variability

**Figure 6:**
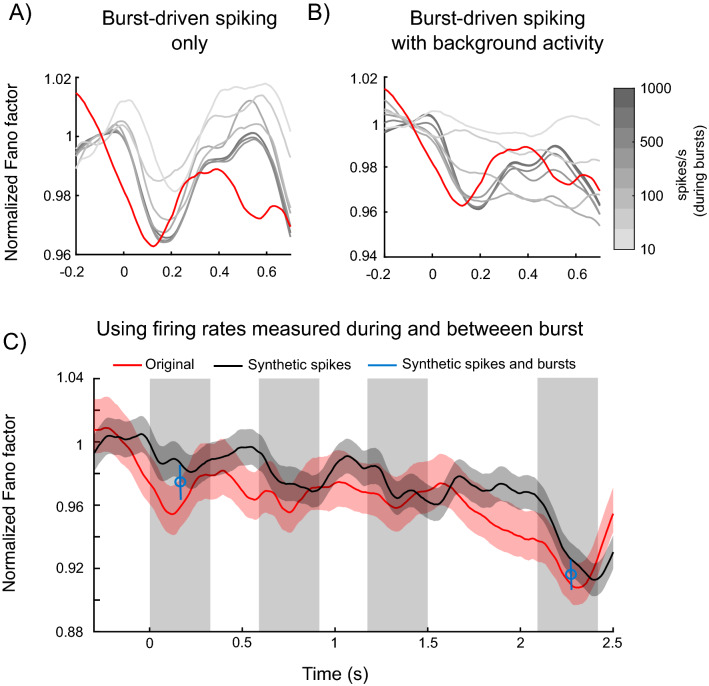
Variability of synthetic spike trains generated based on neurophysiological gamma burst times in Task 1. Spiking rates within the bursts varied from 10 to 1000 sp/s (black to light gray). (**A**) Plotted are the mean (averaged across all the recording sites) FFs normalized divisively by the pre-stimulus average, for a range of simulated spiking rates without (**A**) and with (**B**) an additional synthetic background activity at the level of 1 sp/s. Red line corresponds to the original normalized spike FF for the recorded spiking. (**C**) The FF for original data (red) and synthetic data (black). The synthetic data was created by using the recorded burst times and populating each trial with spikes based on firing rates measured inside and outside bursts per neuron (“Materials and methods” section). Blue marks: FFs in two time windows (normalized by the pre-stim time window; both significantly lower than pre-stimulus window using Wilcoxon rank test, p = 0.007 for sample and p < e−30 for test time window) where both burst counts and the corresponding spiking was synthesized from data. First, burst counts were produced based on the distribution of burst counts in the selected time window for each electrode. The synthetic spike trains were then produced as above (see also “Materials and methods” section).

To further examine the relationship between spiking and gamma burst variability, we used gamma burst events to construct synthetic spike trains. In particular, inside every gamma burst identified (see “Materials and methods” section and Ref.^5^) at every recording site on each trial we generated an artificial Poisson spike train (see “Materials and methods” section). We systematically varied the spike rates inside bursts over a wide range and quantified the resulting synthetic spike variability across trials using FF. The analysis revealed that task-related modulations of the gamma bursts reduced the FF for the artificial spikes to a similar degree as it did for actual recorded spikes (Fig. 6). In fact, this effect was observed across a wide range of spike rates within the bursts, although it was more pronounced for the higher spiking rates. The effect gradually decreased as spike rates inside bursts were reduced to 10 sp/s (corresponding to the average of 0.54 sp/burst given the average burst duration of 54 ms in the data. Thus bursting does not necessarily have to be apparent in single neuron activity.). In addition, we added background spiking activity outside bursts (at 1 sp/s). This resulted in a weakening of the stimulus-driven reduction in spike time variability, especially for low within-burst firing rates (Fig. 6B). This suggests that most spiking has to occur during the bursts for the gamma burst variability to drive the observed drop in spike variability.

In addition, we estimated the average firing rates inside and outside gamma bursts per neuron to generate synthetic spike trains (based on the measured gamma burst times for that neuron). This yielded FFs similar to the ones measured in the data (Fig. 6C, black vs red lines). This suggests that the timing of bursts and the corresponding modulation of firing rates could explain a large part of the reduction in spike variability. The reduction in the variability of the synthetic data was particularly pronounced at periods of increased bursting, during sample and test stimulus onsets.

Finally, we created synthetic burst times (“Materials and methods” section) in three time windows corresponding to the periods before and during the first sample stimulus as well as the first test stimulus. Based on the distribution of burst counts in each time window, we synthesized burst events in epochs corresponding to the three time windows. We then populated those synthetic trial epochs with spikes based on the estimated average firing rates inside and outside bursts for each recorded unit, similarly as in previous analysis of synthetic spike trains. There was a similar reduction in FF from pre-sample to sample stimulus onset as the original data (Fig. 6C, blue marks). Thus, taken together, the reduction in spiking variability during the task can be largely explained by the reduction of gamma burst variability reflecting coordinated population activity.

## Discussion

We found that the task-related modulation of bursts of spiking and gamma power during a WM task resulted in the cross-trial reduction in the variability of neural activity. Further, we found a direct relationship between the reduction of the variability of gamma bursting and the reduction of spiking variability. They co-occurred both in time and space. The reduced spike variability was most pronounced during task events and at recording sites where the reduction in gamma burst FF was large. These were the same sites where gamma bursting and spiking were most strongly task-modulated. However, reductions in activity variability were seen both at recording sites with increased and decreased gamma bursting. Thus, the reduced burst variability was not due to increased bursting per se. This can explain why the reduction in spiking variability is largely decoupled from spike rates^22^. It was the increase in the structure of bursting activity via modulation by the task that produced the reduction in variability. We investigated this by synthesizing spike trains from recorded gamma burst patterns and found that the pattern of bursts could explain reduced spike variability for a wide range of synthetic spike rates including the rates observed in the data. Thus the timing of population bursts before and after task onset can explain a large part of the reduced spiking variability long observed in empirical data^19–23^. It is however important to note that in addition to this, individual neurons have other sources of variability not captured by population bursts, such as stimulus preference or non-Poissonian firing patterns^11^.

These results provide further support for working memory models in which information is held by a combination of spiking (during bursts) and short-term synaptic plasticity (between bursts) rather than persistent spiking alone^15–17,25,27^. In these “synaptic attractor” models bursts of spiking induce temporary synaptic imprints that maintain a trace of the attractor state in between spiking. Further, recent evidence has shown that the short-term synaptic plasticity endows additional benefits beyond memory maintenance. For instance, it helps make recurrent networks more stable and more robust to perturbations and synaptic loss^27,30^.

A recent modeling study argued that intermittent bursting of activity is not compatible with task-related reductions in spiking variability^24^. However, this model used several problematic assumptions that were not in line with experimental observations. Most critically, the Li et al.^24^ model assumed intermittent bursting at random times during the task and they assumed no bursting (i.e., steady spiking) before task onset. Neurophysiological recordings paint a different picture. Task-related bursting is not random. It is highly modulated, strongly waxing and waning with different trial events^5,6^. Here, we demonstrated that this modulation reduces trial-to-trial spiking variability. Neurophysiological recordings, unlike the Li et al.^24^ model, also show bursting before task onset^5,6^. Thus, Li et al.^24^ made comparisons to a baseline with an artificially low level of variability not actually seen in cortex. Finally, it should also be noted that in the Li et al.^24^ model, task-related increases in neural variability were dependent on other questionable model assumptions. Their model could show an increase or decrease in variability depending on whether spike rates change by increasing or decreasing the number vs duration of the bursts (Eq. (9) in Ref.^24^). They chose to increase the duration of the bursts. By contrast, neurophysiological recordings show that burst rates increase but their duration remains constant^5^. We avoided problems with model assumptions by not making any assumptions. Instead, we used data recorded from the PFC of non-human primates.

The reduced variability in spiking and bursting was most prominent during times of elevated bursting—in particular, during encoding of the sample stimuli, the ramp-up of activity towards the end of the memory delays and during test stimulus presentations. The reduction in FF was observed both at sites with neurons actively participating in retention of working memory information and with other, non-coding, neurons. This reduction in variability could boost the fidelity of working memory by increasing signal-to-noise ratio, as has been observed for attention^18,19,31^.

With our ability to record and analyze ever larger quantities of simultaneous neuron activity, there has been increasing evidence for discrete, packet-based activity in cortex^5,32–40^. In this view, underlying cortical processing is discrete events that form packets (bursts) of processed information. It allows time multiplexing such as models of multi-item working memory where each item takes turn being active and silent^15,41^. It would also facilitate inter-areal communication as packets of finite and standardized size are sent and received^33,42–44^. Slow oscillations in the theta and sometimes alpha range have long been proposed to aid such coordinated inter-areal activities^45–47^. These oscillations occur at a similar time scale as the spike bursts and are related^48^. Here we demonstrate that this framework is consistent with task-related reduction in neuron variability that is common across cortex.

## Materials and methods

### Data

We analyzed data from prior studies^5,6,28,29^. This data consisted of three data sets with electrode recordings from PFC while monkeys performed two distinct WM tasks (Task 1, Task2, Task 3: Fig. 1). Task 1 corresponds to 3-item trials in Lundqvist et al ^5^. We focused on 3-item trials as they allowed us to have a larger number of repeated trials. Task 2 corresponds to the recognition task in Refs.^28,29^, which is the same data analyzed in Ref.^6^. Task 3 corresponds to the delayed saccade task in Lundqvist et al ^5^. Task 1 and 2 were recorded with acute electrodes in PFC, yielding both LFPs and isolatable neurons. For Task 3 we had local field-potential data from chronic Utah arrays with no isolated neurons. All the animals received postoperative antibiotics and analgesics and were always handled in accord with the National Institutes of Health guidelines, and all procedures were approved by the Massachusetts Institute of Technology Committee on Animal Care. They were trained with positive reward (juice) only and maintained in accordance with the National Institutes of Health guidelines and the policies of the Massachusetts Institute of Technology Committee for Animal Care). The study is reported in accordance with ARRIVE guidelines.

The LFPs were recorded at 30 kHz in Task 1 and Task 3, and 1 kHz in Task 2. From each task we recorded from two animals (one male Macaca mulatta and one male Macaca fascicularis in Task 1, one male and one female Macaca mulatta in Task 2, two male Macaca mulatta in Task 3). Trial conditions were always randomized between trials. Data was analyzed using automatic scripts, and researchers were therefore blind to group belongings of single trials. The data from Task 1 and Task 3 were down sampled to 1 kHz before further analysis. We kept all electrode contacts with at least one associated isolated neuron in the acute data, all electrodes from the array data. As a result, we obtained 495 units corresponding to 319 electrodes in Task 1 and 233 units on 199 electrodes in Task 2. For Task 3 we had 8 sessions with 64 electrodes in each. Only correct trials were kept for further analysis (on average, 255 +  − 72 trials in Task 1 and 332 +  − 80 trials in Task 2).

Gamma modulated sites were chosen based on activity during sample presentations. To this end, we identified sites with the average gamma burst rate over any of the sample presentations significantly exceeding the threshold of 0.05. The rest of sites were considered as non-modulated.

### Burst analysis

We relied on gamma burst times extracted in the two prior studies^5,6^. In short, we first extracted the gamma-band power in 3 distinct bands (40–65, 55–90 and 70–100 Hz). For this purpose we adopted a multi-taper approach with frequency-dependent window lengths corresponding to 6–8 oscillatory cycles and frequency smoothing corresponding to 0.2–0.3 of the central freq, *f*_0_, i.e. *f*_0_ ± 0.2*f*_0_, where *f*_0_ were sampled with the resolution of 1 Hz (this configuration implies that 2–3 tapers were used). We then extracted an estimate of the band power envelope by averaging spectral components within each band with the temporal resolution of 1 ms. Next, we thresholded the envelopes based on their mean and standard deviation during fixation in pre-stimulus conditions (using the last 300 ms prior to stimulus onset in Task 1, last 500 ms prior to stimulus onset in Task 2).

A gamma burst was defined as a time interval when the envelope exceeded the mean by two standard deviations for at least the duration of 3 oscillatory cycles (using the center of each gamma band to define the length of an oscillatory cycle). In line with our previous work^5,6^ we also used a trial-average measure of burst rates for each spectral band. It corresponds to the chance of a burst occurrence on an individual electrode at a particular time in the trial. In this work, we used the union of bursts across the three gamma bands and, accordingly, we relied on the average burst rate across those identified for the three gamma bands.

### Fano factor (FF)

We estimated the spiking Fano factor (FF) for each unit in a two-step process. First, we calculated the number of spikes, *s*, within a sliding window of 150 ms and calculated its mean, *μ*_*s*_, and variance, *σ*_*s*_^2^, across trials to estimate FF = *σ*_*s*_^2^/ *μ*_*s*_. This resulted in a raw FF time series of 1 ms resolution. Further, to facilitate comparisons we show in Figs. 2 and 3 the relative FF, which is obtained by removing the mean FF over the pre-stimulus baseline from the raw FF.

The FF for bursts was estimated using a binary time series for each site and trial with 0 corresponding to the time bin outside a burst event and 1—inside a burst event. The resulting time series was then subject to the same treatment as spike time series.

### Synthetic spike train generation

We constructed synthetic spike trains based on the recorded gamma burst timings. In particular, we randomly placed spikes inside and outside burst intervals for each corresponding trial and channel to match the desirable target average firing rates inside and outside bursts. For the results shown in Fig. 6A, the average firing rates inside bursts were systematically varied from 10 to 1000 sp/s (with no spikes outside burst intervals). In another manipulation (Fig. 6B) we added background spike activity with the constant rate of 1 sp/s in each trial. Finally, following our approach for gamma burst dependent random spike generation, we produced synthetic spike trains with the target firing rates inside and outside bursts corresponding to the average firing rates in the recorded data inside and outside bursts, respectively (Fig. 6C). Importantly, these averages were estimated (and then used for spike generation) independently for each channel, i.e. burst events for each electrode were matched with spiking units measured on that electrode. In addition, in three 150 ms windows (− 250 to 100 ms before stimulus onset, 75 to 225 ms after stimulus onset, 75 to 225 ms after test onset) we used the recorded burst count distributions to create synthetic burst events. We then populated these synthetic bursts with synthetic spikes and calculated corresponding FFs.

## Data Availability

The datasets used in the current study are available from the corresponding author on reasonable request.
